# The complications of male hypogonadism: is it just a matter of low testosterone?

**DOI:** 10.3389/fendo.2023.1201313

**Published:** 2023-06-28

**Authors:** Elisabetta Veronica Munari, Myriam Amer, Alessandro Amodeo, Ruggiero Bollino, Silvia Federici, Giovanni Goggi, Luca Giovanelli, Luca Persani, Biagio Cangiano, Marco Bonomi

**Affiliations:** ^1^ Department of Medical Biotechnology and Translational Medicine, University of Milan, Milan, Italy; ^2^ Department of Endocrine and Metabolic Diseases, IRCCS Istituto Auxologico Italiano, Milan, Italy

**Keywords:** osteoporosis, endothelial dysfunction, cardiovascular events, hypogonadism, complications

## Abstract

The history of diagnosing hypogonadism and hypotestosteronemia shows us the many steps that were necessary to achieve our current knowledge and the ability to improve these patients’ well-being. Moreover, so far, criteria for diagnosing hypotestosteronemia varies according to the underlying condition, and according to the consensus or guideline adopted. Furthermore, besides the many signs and symptoms, there are several complications associated with low testosterone levels such as osteoporosis, metabolic alterations, as well as cardiovascular disorders. However, data are often conflicting regarding the severity, timing or even the real clinical relevance of these complications, although these studies often lack essential information such as gonadotropin levels or the underlying cause of hypogonadism. The present review focus on the complications of male hypogonadism according to the cause of testosterone deficiency, highlighting the lack of information found in many studies investigating its effects. We thereby stress the necessity to always perform a complete evaluation of the type of hypogonadism (including at least gonadotropins and secondary causes) when investigating the effects of low testosterone levels.

## 1 The history of hypogonadism

The history of medicine is dotted with great intuitions leading to therapeutic advancement and increase in patient survival and/or well-being, and the discovery of hypogonadism as distinct condition, determining complications and requiring medical care, makes no exception. A long time has passed since one of the earliest experiments involving testicular hormones was carried out in 1849 by Arnold A. Berthold who observed that testes transplanted from roosters to capons restored androgenic functions in the latter group (1); he was, in fact, the first to postulate a humoral effect of testes on distant organs as a general principle concluding that “the testes act upon the blood, and the blood acts upon the whole organism”, expressing a key concept in Endocrinology (2). Forty years later, Brown-Séquard (3) was reporting results on self-injections of testicular extracts, stating that “his vigor and feeling of well-being were markedly restored but the effects were transient”. He was thus suggesting the extract contained a biochemical substance that promoted rejuvenation, thus resulting in the first man to ever resort to doping, albeit without obtaining any benefit in sport competitions since he was then 72 years old. Still, he reported a great boost in “sexual prowess”, even though this achievement, soon imitated by many, was probably due to a placebo effect. Furthermore, as the psychological implications of the experiment increased his manly behavior, also gave a boost to the research in the endocrinological field. The dice was cast: in 1935 the Organon group published “On Crystalline Male Hormone from Testicles (Testosterone)” and were the first to describe the isolation of the hormone, which was named *testosterone*, from the stems of *testicle* and *sterol* and the suffix of *ketone* (4). In the same year Butenandt and Hanisch (5, 6) were successful in the chemical synthesis of testosterone (T) from cholesterol followed by L. Ruzicka and A. Wettstein, from the Ciba group in Zurich, who published their synthesis of testosterone (5). And yet, the diagnosis of hypogonadism for centuries was largely made on clinical grounds and was known to be caused by several medical etiologies, including pituitary tumors and absent or atrophic testicles. However, soon after the availability of testosterone preparations, it was already recognized that hypogonadism could exist without these major, identifiable causes. The modern history of testosterone deficiency and its replacement therapy arguably begins with the introduction of the radioimmunoassay methodology (RIA) into clinical medicine in the 1970s; indeed, this was the birth of modern Endocrinology. The introduction of the RIA methods, in fact, shifted the diagnosis of testosterone deficiency from signs and symptoms to an undue emphasis on blood tests results. The availability of a quantitative method to investigate these clinical conditions, and to measure the hormone deficiency gave a newfound certainty to the physicians in their practice, and the possibility to accurately research this condition. Nonetheless, with research came the knowledge of the side effects of high testosterone serum levels, and the assertion by Huggins and Hodges in 1941 that testosterone activated prostate cancer (PCa) (6) threw a shadow for the next 70 years. Indeed, the fear of PCa was the primary deterrent to the use of testosterone therapy for decades. Prescription rates increased as accumulated evidence showed testosterone therapy was not associated with increased new PCa risks. The observation that androgenic stimulation of PCa reaches a maximum at relatively low testosterone concentrations - the saturation model - provided the theoretical framework for understanding the relation between androgens and PCa and led to multiple case reports leading to reassuring results of testosterone therapy in men with PCa. In 2016 the Testosterone Trials provided high-quality evidence of multiple benefits of testosterone therapy, nearly all of which had been recognized by clinicians by 1940 (2).

## 2 The many faces of the diagnosis of male hypogonadism

The clinical presentation of patients with deficient testosterone production or action depends on the age of onset of hypogonadism; androgen deficiency during the third trimester of pregnancy and during the minipuberty, may cause defects in testicular descent leading to cryptorchidism as well as micropenis (7–9). Postnatal prepubertal testosterone deficiency leads to poor secondary sexual development and eunuchoid skeletal proportions (10–12). Instead, if testosterone deficiency develops after puberty, the patient may complain of decreased libido, erectile dysfunction, and low energy. The current guidelines of the Endocrine Society recommend diagnosing hypogonadism in men with symptoms and signs of testosterone deficiency and unequivocally and consistently low serum total testosterone and/or free testosterone concentrations. However, how this apply in different forms of hypogonadism is not as consistent as we would like it to be. In particular, the abovementioned guidelines use a total testosterone cutoff of 264 ng/dL (9.2 nmol/L) to define a biochemical androgen deficiency, in healthy nonobese young men (13). Of course, they also prompt measuring total testosterone on two different mornings while the patient is fasting, as serum testosterone concentrations vary significantly because of diurnal, circadian, and circannual rhythms, episodic secretion, and assay variations, and they also exhibit a diurnal variation with peak values in the morning; aging reduces the magnitude of this diurnal variation (13). Still, the diagnosis of hypogonadism is not universal: in fact, it can vary depending on the type of hypogonadism considered and the different specialists who diagnose it. In fact, many other different cutoffs have been used and suggested for the diagnosis of different forms of hypogonadism as it is shown in **Table 1**; for example, a very low total testosterone levels (below 3.5 nmol/L or 100 ng/dL) are often used to define congenital hypogonadotropic hypogonadism (CHH), even though some evidence suggest that milder forms could have the same origin (14). When considering acquired and functional forms of hypogonadism, the EMAS group defined late-onset hypogonadism (LOH) by the presence of at least three sexual symptoms (decreased sexual interest and morning erections and erectile dysfunction) associated with a total testosterone level below 11 nmol/liter (302 ng/dL) and a free testosterone level of less than 220 pmol/L (64 pg/mL). Similarly, the EAU (European Association of Urology) guidelines define the total testosterone threshold of 12.1 nmol/L and recommend measurements of free testosterone in men with total testosterone levels close to the lower normal range (8 - 12 nmol/L, the “grey area”) or abnormal SHBG. The AUA (American Urological Association) guidelines, instead, define the total testosterone threshold of 10.4 nmol/L and don’t recommend routine use of free testosterone (15).

**Table 1:** Guidelines for the diagnosis of late-onset hypogonadism (LOH).

Guideline	Total testosterone nmol/L (ng/mL)	Calculated free testosterone pmol/L (pg/mL)	Testosterone assessments required	Questionnaire
American Urological Association	10.4 (3.0)	NA	2 times	Not recommended
British Society for Sexual Medicine	Mild: 12.1 (3.5) Severe 8.0 (2.31)	NA 225 (65)		
Canadian Endocrine Society	Depending on reference values in local laboratory	NA		
European Association of Urology	12.1 (3.5)	243 (70)	2 times	Not recommended
Endocrine Society	9.2 (2.64)	NA		
International Society for Sexual Medicine	12 (3.5)	NA		
International Society for the Study of the Aging Male	12.1 (3.5)	243 (70)	Not suggested	Recommended
European Society of Endocrinology and European Academy of Andrology	11.1 (3.2)	220 (63)	2 times	Not recommended

NA, not available.

Of course, once that hypogonadism has been diagnosed (despite these variable cutoffs available), it is very important to measure gonadotropins (LH and FSH) levels to differentiate primary from secondary forms. In **Table 2** are reported the causes of hypogonadotropic and hypergonadotropic hypogonadism. It must be noted that among secondary acquired hypogonadisms, are listed the functional forms, associated with different diseases and drugs; these forms are often considered as a whole in its management, even if the EMAS study has clearly shown that LOH can often be both hypogonadotropic and hypergonadotropic, that cannot obviously be overlapping conditions due to pathogenesis.

**Table 2:** Classification of hypogonadism and common causes of primary and secondary hypogonadism.

Central Hypogonadism		Primary Hypogonadism	
Functional	Organic		
• Chronic illness • Malnutrition • Excessive exercise • Stress • Drugs (e.g. opioids, anabolic steroids, glucocorticoids) • Alcohol and marijuana abuse • Other endocrine disorders (e.g. hyperprolactinemia, hypothyroidism) • Severe obesity • Some sleep disorders (e.g. OSAS) • Organ failure (kidney, heart, and lungs)	• CHH (normosmic CHH; Kallmann’s syndrome) • CHARGE syndrome • CHH with CAH • MPHD • Hypothalamic-Pituitary region lesions (e.g. craniopharyngioma) • Metabolic diseases (e.g. hemochromatosis) • Hypophisitis • Infiltrative diseases • Thalassaemia • Infections • Inflammatory diseases (e.g. Langerhans Cell Histiocytosis) • Granulomatous disease (e.g. sarcoidosis) • Iatrogenic causes (e.g. radiotherapy) • Other genetic syndromes (e.g. Prader-Willi, Laurence Moon Biedl etc)	• Klinefelter syndrome • Tuner syndrome • Anorchia • Enzymatic defects • DSD (e.g. gonadal dysgenesis) • LH/FSH resistance • Acquired forms (e.g. chemo- and/or radiotherapies, androgen synthesis inhibitors, autoimmune diseases, end stage renal disease, trauma, gonadal torsion, varicocele) • Advanced age	
	**Frequencies**		
20% Male 20% Female	10% Male 20% Female	5-10% Male 25% Female	

CHH, congenital hypogonadotropic hypogonadism; CAH, congenital adrenal hypoplasia; MPHD, multiple pituitary hormone deficiencies; DSD, disorders of sexual development.

### 2.1 Complications related to male hypogonadism

Other than the alteration of the sexual function, low testosterone levels may lead to several clinical implications.

Hypogonadism is a well-established cause of secondary osteoporosis in men. Interestingly, the percentage of hypogonadism found in male osteoporosis has been shown to be around the 15%; however, this finding is not an absolute value and can differ based on the definition of hypogonadism and the population considered (16–20).

Nonetheless, reduced androgen levels are associated with low bone mineral density, increased markers of bone turnover, and increased risk of fracture. In adults, hypogonadism can lead to loss of bone mass, while before puberty it can cause failure to reach bone mass peak (21–24).. Testosterone affects the skeleton in several ways. First, androgens contribute to bone size development through their effect on periosteal apposition, and this explains the larger and wider bones in men compared with women (25). Larger bones are associated with better bone geometry, which results in increased bone strength. Second, testosterone is the main source of estradiol (E2) in humans through aromatase activity, and E2 is a key regulator of bone metabolism (25). Third, testosterone has a direct anabolic effect at the bone level by stimulating osteoblast differentiation and proliferation (25). Indeed, androgen receptors have been identified on various bone tissue cells, particularly osteoblasts, osteoclasts, and mesenchymal stromal cells. Fourth, not to be forgotten, is testosterone action on the PTH–vitamin D axis, in fact low levels of T imply decreased action of renal 1a-hydroxylase and consequently of the active 1,25-(OH) vitamin D concentration (26). Despite the well-established correlation between hypogonadism and altered bone metabolism, the testosterone level below which skeletal consequences begin to develop has not been identified yet. Some studies suggest that bone depletion is greatest when E2 concentrations below 10 pg/mL and/or testosterone levels below 6.94 nmol/L are found (27, 28).

Men with androgen deficiency are also characterized by decreased physical energy and motivation, presence of depressed mood and irascibility, sleepiness and decreased attention and memory (13). If the androgen deficiency is severe, erythropoiesis is highly reduced, and this can lead to a mild hypo-proliferative normocytic, normochromic anemia. The long duration of testosterone deficiency also brings about a reduced muscle strength and thus of physical and work activity because of reduction in muscle bulk. Moreover, hypogonadism is associated with higher body fat, central obesity worse metabolic profile and higher risk of diabetes and metabolic syndrome (29).

The relationship between low testosterone and diabesity, defined as the coexistence of obesity and altered glycemia (comprising insulin resistance, the metabolic syndrome and type 2 diabetes) is bidirectional, although the evidence suggests a stronger effect of diabesity on hypothalamic-pituitary-testicular axis suppression compared with the effect of hypogonadism on promoting diabesity (30). Potential mechanisms by which low testosterone leads to altered glycemia in men have been identified. Testosterone plays a role in metabolically favorable changes of body composition, increasing lean mass and decreasing fat mass, but also body composition-independent effects have been reported (31). Androgens stimulate myogenic differentiation and inhibit adipogenesis (32), increase catecholamine-induced lipolysis (33) and augment insulin sensitivity in adipose tissue and muscle (34). However, testosterone may also regulate insulin sensitivity in a direct and acute way before changes in body composition are expected to occur (35).

Even if in the past an alert emerged concerning cardiovascular risks due to testosterone treatment, these worries have today been questioned, especially when the treatment is performed with medical supervision. The studies that found an association of testosterone treatment with CV events had in fact some major biases, including lack of T measurement during follow-up, treatment inadequacy, comparison with patients treated with phosphodiesterase type 5 inhibitor (which are associated with cardioprotective effects), lack of proper randomizations and broad definition of CV-related events which even included self-reported minor events (36). On the other hand there are no published long-term placebo-controlled studies whose primary goals are to evaluate the effect of TRT on the cardiovascular system (37). Moreover, even if a sub-study showed a greater increase in coronary artery non-calcified plaque volume in patients during TRT compared to placebo group (38), the United States Testosterone Trial, the largest placebo-controlled trial of testosterone therapy in older men to date, did not demonstrate increased cardiovascular events after one year of TRT compared to 186 placebos (39). Thus, no definitive evidence is available about TRT effect on cardiovascular outcomes in frail men or those at higher risk of a cardiovascular event (38).

On the other hand, studies have sustained negative relationship between low serum T levels and increased risk of cardiovascular disease, and both cardiovascular and all-cause mortality (40, 41). Other studies have indeed suggested that both ED and testosterone status may independently predict subsequent CVD-related events and mortality, particularly in the presence of cardiometabolic risk factors (42, 43), possibly due to systemic inflammation which participates in the activation of innate and adaptive immune cells and contributes to tissue damage, atherosclerosis, and insulin resistance, together with the other mechanisms. Furthermore, the role of testosterone as arterial vasodilator within the coronary circulation and other vascular beds is well known (44). However recent observational research lasting more than 12 years found that ED, independently of serum T, predicted all-cause mortality (45).

The occurrence of all these complications, and the patient’s overall quality of life, is more pronounced in men with severe or moderate forms of LOH and appear to be less pronounced in patients with low testosterone levels without sexual symptoms (27).

### 2.2 Is it just hypotestosteronemia?

Many of the studies evaluating the complications of hypotestosteronemia, in addition to the different cutoffs used to diagnose hypogonadism, often also fail to investigate the type of hypogonadism, if the clinical outcomes depend solely on testosterone. But how can we be sure that this is really the case? We know that very different condition according to pathogenesis, cause, and comorbidities can produce hypotestosteronemia; so, how can we separate the effects of testosterone and of the underlying condition?

### 2.3 Klinefelter syndrome

As it is widely known KS is the most common X-chromosome related condition (with the chromosomal pattern that ranges from classic 47 XXY to mosaic forms such as 47, XXY/46XY up to more complex polysomies such as 48XXXY or 48XXYY) and also a frequent cause of hypogonadism in men, presenting with an incidence among newborns of 1:500 to 1:1000 (46). It is characterized by a wide spectrum of heterogeneity in its clinical and genetic presentation, and it is common knowledge that many KS features are testosterone independent.

Among these patients the inactivation of the supernumerary X is not complete, and a portion can be spared from inactivation (47), as it normally happens in women, and this may also explain the tendency to gynoid proportions or female prevalent conditions (e.g., breast cancer, and autoimmune diseases) (48). A portion of the gene that undergo inactivation is the androgen receptor gene (*AR*). Some clinical research studies have found a correlation between AR sensitivity and anthropometric parameters in KS individuals (48–50), although others have also claimed that a clear proof is still lacking (51). Moreover, there are portions of sex chromosomes that do not undergo X inactivation named pseudo-autosomal regions (PAR 1 and PAR2) (52) resulting in three active copies in KS. The gene located on PAR1 is Short-stature Homeobox on chromosome X (SHOX), which likely determines the tall stature and long legs associated to the clinical picture of KS patients (53). Moreover, not all the complications of KS are due to the low testosterone levels, as shown in **Table 3**. For example, KS patients present 5 times higher prevalence of metabolic syndrome (MetS) based on epidemiological studies compared to the age-matched controls (54, 55), which is still higher when comparing KS patients to the ones with hypogonadism for other causes (46% vs. 27%) (56). The altered metabolic profile found among KS subjects gives an increased prevalence of diabetes mellitus (DM) type 2 and a tendency to develop thrombosis/embolism, both of which are cardiovascular (CV) risk factors (57). Furthermore, the dyslipidemia, with both high level of total and low-density lipoprotein (LDL) and triglycerides (58, 59), associated with a reduction of HDL (60), is not modified by testosterone treatment (61) suggesting an independent effect from testosterone (62).

**Table 3:** Comorbidities in KS patients attributed to supernumerary X.

Comorbidities in KS patients attributed to supernumerary X
Neurocognitive deficits (e.g. spoken language disorders, intellectual disability, executive and social dysfunction)
Neurologic disorders (e.g. epilepsy, seizures, and tremor)
Metabolic disequilibrium (e.g. decreased lean mass, increased abdominal fat deposition, insulin resistance and type 2 diabetes)
Osteopenia/Osteoporosis and related fractures
Gynecomastia
Cryptorchidism and small testes
Infertility (e.g. severe oligospermia or cryptospermia)
Thrombosis (e.g. recurrent venous ulcers, venous insufficiency, deep venous thrombosis and pulmonary embolism)
Congenital heart defects (e.g., mitral valve prolapse, atrial and ventricular septal defects with patent ductus arteriosus, hypoplasia of the bilateral internal carotid arteries and dilatation of the bilateral vertebral arteries with weakness of the limbs, hypertrophic cardiomyopathy, and partial anomalous pulmonary venous connection with pulmonary arterial hypertension)
Neoplasia (e.g. breast cancer, hematological neoplasms, extragonadal germ cell tumors, Leydig cell tumors, lung cancer)
Autoimmune disorders (e.g. Addison’s disease, diabetes mellitus type 1, multiple sclerosis, acquired hypothyroidism, systemic lupus erythematosus, rheumatoid arthritis, Sjogren’s syndrome)
Ophthalmologic problems (e.g. retinal dysfunction, impaired day vision/night vision
Dental problems (e.g. taurodontism, caries)

KS subjects have also higher prevalence of congenital heart disease independent from testosterone (63, 64), with relative increased mortality (65). KS patients are also at increased risk of venous thromboembolism (66), and pulmonary embolism (PE) (63, 67).

Among these individuals there is as well as an increased risk for arterial thromboembolism (65), with an increased expression of thrombin generation both in platelet-poor plasma (PPP) and platelet-rich plasma (PRP), partially related to increased FVIII levels explaining their procoagulant diathesis (68).

Finally, among KS patients the increased risk of developing osteoporosis and osteopenia and in the worst cases, clinical fractures (69, 70),, especially if the diagnosis of KS is made at a higher age (70), seems not completely dependent on hypotestosteronemia. KS decreased bone mass in 25–48% of cases (71) and osteoporosis in 6–15% (72), is due to decreased bone deposition and increased bone resorption (73–75). Still, some studies have considered bone microarchitecture in addition to the volumetric BMD, and estimated bone strength of the radius and the tibia, by using quantitative computed tomography (pQCT). The results confirmed a reduced tibial cortical area and reduced trabecular density which overall can negatively influence the estimated bone strength (76, 77). The increased risk of osteoporotic bone fractures, as observed in the available epidemiological studies (63) could be therefore also due to this worse bone quality to the reduction of BMD (69, 78).

The androgens deficiency have of course a role in the reduction of bone density and development of osteoporosis in KS (79), but also KS patients with normal T levels have a reduced bone mass (80), and, is controversial if it is possible to reverse the decreased bone mass found in KS men with TRT (60, 71, 81, 82). Possible other player on bone mass other than testosterone in KS are an increased aromatization of T into E2 (73, 83), very low vitamin D levels (63, 84, 85), and the disproportionate ratio fat/muscle (86). Nevertheless, a genetic component cannot be excluded (84, 87). Evidence have clearly identified the association of the length of AR sensitivity (and CAG polymorphism length) with the inactivation through methylation of the two X chromosomes (48, 88–91) with a nonrandom X inactivation and lower androgen activity, especially in case of KS with normal T concentration. In the **Table 4** there is a summary of all the possible contributors of reduced bone mass in KS. Together, they can be responsible for the pathogenesis of early bone mass loss in KS from the end of puberty onward. However, it remains obscure the treatment of choice in eugonadal osteoporotic KS patients. In the future, pharmacologic studies on AR sensitivity and the possible elaboration of drugs with similar action of INSL3 could be of particular interest.

**Table 4:** Probable players involved in the reduced bone mineral density in KS patients.

Whole bone	Cancellous bone	Compact bone
Altered body composition (disproportionate FMR)	Nonrandom X chromosome inactivation and AR CAG length	Reduced testosterone and estrogen levels
Hypovitaminosis D	Androgen resistance (reduced AR expression)	Combined AR and estrogen receptor disruption
	Reduced androgens levels	
	Reduced INSL3 levels	

FMR, fat-to-muscle ratio; AR, androgen receptor; INSL3, insulin-like factor 3 levels

### 2.4 Hypergonadotropic and hypogonadotropic hypogonadism and bone

As said, in the past years, follicle stimulating-hormone (FSH) has been shown to stimulate bone resorption, by binding directly to osteoclasts (92, 93). It has now been a decade since the hypothesis was put forward that not only estrogen levels in women may cause reduction in bone mass, but that FSH may also play a direct role; in fact, several observational studies involving a large number of perimenopausal and postmenopausal women reported an association between high FSH values and increased bone turnover, as well as reduced bone mineral density (BMD) (94, 95). In addition to these findings, it has been noticed that in younger women, hypergonadotropic amenorrhea was found to be associated with greater bone loss than hypothalamic amenorrhea, regardless of serum estradiol levels (96). It must be said that the data are far from univocal, and if another study trying to find an inverse correlation showed that FSH concentrations could be a good negative predictor of BMD in women on sex replacement therapy (97), other two studies have shown no relevant relationship between FSH and bone metabolism (98, 99). A very recent cross-sectional observational study investigated the possible role of FSH excess also in male bone health by comparing, for the first time, in a cohort of 119 men, primary and central hypogonadism (100). In this study only patients who had never undertaken testosterone therapy with spontaneous pubertal development were enrolled; they were divided into three groups according to their FSH levels: hypogonadotropic hypogonadism, hypergonadotropic hypogonadism and Klinefelter syndrome (KS) patients, who were distinguished from the other forms, based on the onset of FSH elevation, thus making it possible to understand the different effects of various forms of hypogonadism on bones. After adjusting for potential confounders, the study found that patients with hypergonadotropic hypogonadism not caused by KS showed significantly lower BMD at lumbar spine and tended to show lower BMD at femoral neck, as compared to those with hypogonadotropic hypogonadism. Also, in KS men BMD at lumbar spine was significantly lower than in those with hypergonadotropic hypogonadism not caused by KS. Thus, enforcing the notion of a direct effect of FSH excess and its duration, even in the male subjects on bone metabolism demineralization (100).

### 2.5 Endothelial dysfunction and hypogonadotropic hypogonadism

Circulating endothelial progenitor cells (EPCs) are bone marrow-derived cells required for endothelial repair. Since endothelial dysfunction is known to be the first step of the atherosclerotic process, a low EPC number can be considered as an independent predictor of endothelial damage, future cardiovascular events (101) and also death from cardiovascular cause (102).

Several studies have shown that patients with hypogonadism without other confounding risk factors have a low number of circulating progenitor cells (PCs) and EPCs (103). When it was investigated if testosterone replacement therapy could increase circulating EPC number in men with hypogonadotropic hypogonadism (HH), it was shown that, even though serum total testosterone levels before replacement therapy were not significantly associated with the number of circulating EPCs in men with late onset hypogonadism, testosterone replacement therapy (TRT) could increase their number, implying a benefit of TRT on endothelial function in hypogonadal men (104). Similarly, another study evaluated the levels of PCs and EPCs in men with HH after prolonged testosterone replacement therapy finding that hypotestosteronemia was associated with a low number of circulating PCs and EPCs in young HH subjects and that testosterone treatment was able to induce an increase in these cells, thus suggesting a possible direct effect on the bone marrow (103). Other evidence of the association of HH with endothelial dysfunction, comes from a very recent study which has shown that in these patients there is an increased oxidative stress, measured as oxidation protein products (AOPP), total antioxidant capacity (TAC) and total oxidant status (TOS), that is at least partially reduced by testosterone replacement therapy (105).

It must be noted that all these studies are performed in patients with hypogonadotropic forms with an astounding lack of results concerning primary testosterone deficiencies, leading to a void of knowledge on whether these alterations are actually due to just testosterone levels (as suggested by the partial reversal in exogenous treatment) or are in part caused by the underlying origin of hypotestosteronemia, such as metabolic conditions (which are known to be related with pro-atherogenic conditions). The only exception is one study done on patients with Klinefelter syndrome (KS), but as already mentioned, this condition must be considered apart from other testicular failures. In this study, KS patients were compared with healthy controls and both groups were further divided according to the presence or absence of cardiovascular risk factors. Based on the results obtained, authors speculated that factors involved in KS, whether hypogonadism, cardiovascular risk factors or other genetically determined factors related to the supernumerary X chromosome might contribute to a reduction in EPCs number and that this could be considered another cardiovascular risk factor contributing to the increased mortality of these subjects (106). Thus, we cannot know if there is a predisposing condition to hypotestosteronemia that also causes endothelial dysfunction.

## 3 Conclusions

Of a total of 166 articles evaluated in this review, an alarmingly low number of only 74 used the gonadotropin assay in the diagnosis of hypogonadism. Based on what it has been said so far, hypogonadism is not a single disease but a galaxy of distinct conditions that can sometimes be both cause and consequence of low testosterone levels. The lack of consistency not only in the diagnosis of hypogonadism, but also in the information provided by the authors while studying its complications, hinders the understanding of the consequences of testosterone alone and those of the different types of hypogonadism, preventing the understanding necessary for the correct management of each of these diseases. Future studies on hypogonadism should therefore increasingly be directed toward discovering the cause based on the type of hypogonadism, whether it is hypogonadotropic or hypergonadotropic, whenever studying its complications, always assessing gonadotropins and secondary causes.

## Author contributions 

EM, MA, BC, and MB contributed to the conception and first draft of the manuscript. EM, MA, AA, and RB collected clinical data. BC and MB, contributed to the supervision and critical revision of the manuscript. All authors contributed to the article and approved the submitted version.
